# Radiofrequency Ablation for Chronic Hip Pain: A Case Report on the First Application in Morocco

**DOI:** 10.7759/cureus.80814

**Published:** 2025-03-19

**Authors:** Khaoula Kassi, Manare Jaai, Ahmed amine El Oumri

**Affiliations:** 1 Physical Medicine and Rehabilitation, Faculty of Medicine and Pharmacy, Mohammed VI University Hospital Center, Oujda, MAR

**Keywords:** articular branch of the femoral nerve, articular branch of the obturator nerve, chronic hip pain, failure of radiofrequency ablation, hip osteoarthritis, hip pain management, osteonecrosis of the femoral head, radiofrequency ablation procedure

## Abstract

Hip joints are vulnerable to various disorders that can lead to severe, chronic pain. Conventional treatments typically offer only temporary relief. For patients who are not candidates for hip prostheses, radiofrequency (RF) nerve ablation may present a promising alternative.

A 60-year-old man with stage 5 osteonecrosis of the femoral head presented with severe chronic hip pain. After declining total hip replacement, he underwent RF nerve ablation, resulting in a modest 20% reduction in pain.

The limited effectiveness of RF nerve ablation is partly due to insufficient knowledge of hip joint innervation and nerve regeneration. In this case, advanced osteonecrosis further complicated the procedure by obscuring anatomical landmarks. Consensus guidelines and clear protocols for using radiofrequency ablation (RFA) in chronic hip pain are essential for improving treatment outcomes.

## Introduction

Hip joints are vulnerable to various disorders that can lead to severe, chronic pain. It is estimated that 10% of individuals over 40 will experience hip pain [1]. While conservative treatments, such as physical therapy, lifestyle modifications (e.g., weight loss), pain-relieving medications, and intra-articular injections, are commonly employed, they often provide only temporary relief and may have significant adverse effects [2]. Although hip prosthesis is a definitive treatment for many, not all patients are suitable candidates or may choose to avoid surgery. Furthermore, persistent pain affects 7%-23% of patients after total hip arthroplasty [3]. In such cases, nerve ablation using radiofrequency (RF) methods may offer a promising alternative. This case report explores the application of this technique and evaluates its potential benefits.

## Case presentation

**Figure 1:**
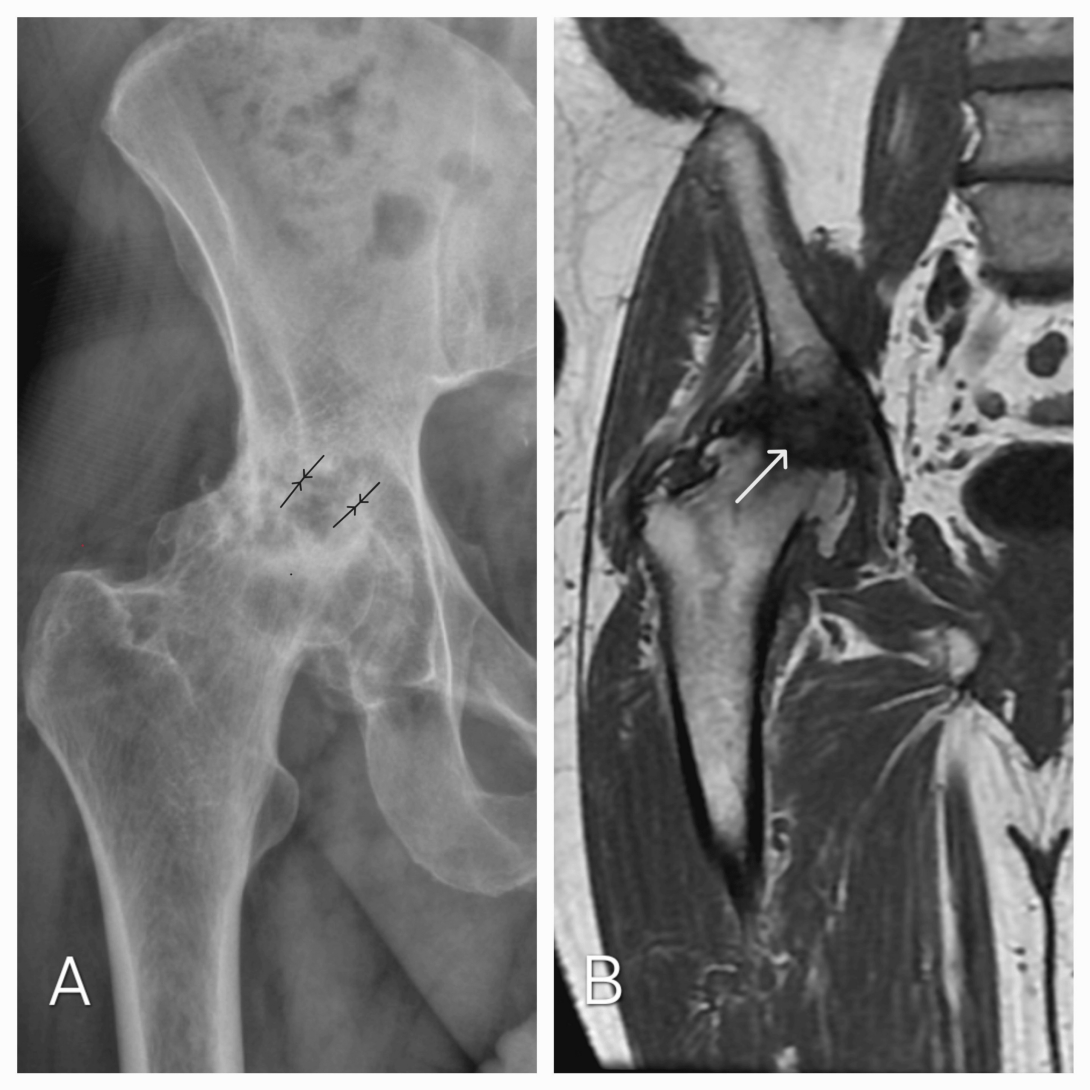
MRI and X-ray showing osteonecrosis of the femoral head (stage 4) (A) X-ray: severe coxofemoral joint narrowing (arrows) with subchondral cysts and femoral head flattening. (B) MRI: coronal view showing the hypointense necrotic area (arrow), deformity of the right femoral head, loss of sphericity, and coxofemoral joint narrowing.

A 60-year-old man presented to the physical medicine and rehabilitation department for the management of hip pain due to idiopathic osteonecrosis of the femoral head, after refusing total hip replacement. He developed right groin pain six months ago, which was mechanical, aggravated by walking and relieved by rest. The pain was continuous, non-radiating, and scored 7/10 on the visual analog scale (VAS). It was not improved by non-steroidal anti-inflammatory drugs or paracetamol. The patient had no history of smoking, alcohol use, corticosteroid use, or trauma. The physical examination revealed normal body mass index (BMI = 23.4), with a limited range of motion in the right hip joint (reduced internal rotation, external rotation, extension, and flexion) and quadriceps atrophy. The contralateral side was assessed as normal, and the patient presented with a limp (walking with crutches). To evaluate physical disability, we used the Western Ontario and McMaster Universities Osteoarthritis Index (WOMAC). The patient's WOMAC score was 58, indicating severe symptoms. X-ray and magnetic resonance imaging confirmed stage 4 osteonecrosis of the femoral head, according to the Association Research Circulation Osseous (ARCO) classification (Figure 1). 

After the patient refused and failed to achieve pain relief with first- and second-line analgesics, radiofrequency ablation (RFA) of the articular nerves was recommended. This decision followed a prognostic test involving ultrasound-guided nerve blocks of the obturator nerve (ON) and femoral nerve (FN), using 0.5 mL of 0.5% bupivacaine at the FN site and 0.75 mL at the ON site. The patient was positioned supine on the fluoroscopy table. The skin over the hip was disinfected with chlorhexidine twice and draped with sterile towels. Hip puncture sites were anesthetized with a local 2% lidocaine injection. To lesion the articular branch of the FN, an electrode was inserted below the anterior superior iliac spine, with the electrode tip positioned below the anterior inferior iliac spine, near the anterolateral margin of the hip joint (toward the 12 o'clock position of the acetabulum). For the obturator nerve, the needle was inserted medially into the femoral artery, just below the inguinal ligament. The electrode was placed beneath the inferior junction of the pubis and ischium, which appears teardrop-shaped in the anteroposterior radiograph. Sensory stimulation was performed at 50 Hz up to 0.8 V to localize the pain area, followed by a motor test at 2 Hz up to 2.5 V. After confirming the absence of motor response, the neurectomy was performed at 80 °C for 120 seconds at each site. Following the procedure, the needles were withdrawn, and sterile bandages were applied to the RFA sites.

Outcomes

Three months after the procedure, the patient reported minimal pain relief, with a VAS reduction from 7/10 to 5/10, and no improvement in the WOMAC score. No adverse effects were observed.

## Discussion

RF techniques in chronic pain management are used to modulate sensory nerve transmission, typically by producing thermal lesions [4]. The indications for RF treatment in chronic hip pain are not well-established. RF is generally used when conservative treatments fail for chronic articular pain, regardless of whether the pain is due to degenerative, inflammatory, or tumoral conditions. However, the effectiveness of RF treatment for each specific pathology remains unclear. No medical society guidelines currently exist for patient selection for hip articular branch RF [5]. To differentiate articular pain from radiating pain (e.g., due to spinal diseases), nerve blocks or intra-articular corticosteroid injections are used, with a sensitivity of 87% and a specificity of 100% [1]. These tests also provide prognostic information on the likely success of RF treatment. In our case, with the articular origin confirmed, we used the nerve block as a prognostic test.

All studies targeting the articular branches of the ON and FN have focused on these branches, with most targeting the FN articular branch [2-5]. The femoral and obturator articular branches are the primary sources of innervation to the hip capsule [1], making ablation of both recommended [6]. Fluoroscopy is essential for accurately targeting these branches. The landmark for the ON articular branch is just below the "teardrop" at the pubic and ischial junction, while for the FN articular branch, it is just below and medial to the anterior inferior iliac spine, toward the 12 o’clock position of the joint acetabulum [6]. Some studies also use ultrasound to avoid penetrating the femoral neurovascular bundle, although the added safety or efficacy of ultrasound guidance remains unclear [5]. Once the lesion points are located, a motor test should be performed [6]. Neuro-ablative temperatures range from 75 to 90 °C for 60-300 seconds [2-5].

Favorable results have been observed in the majority of studies. In the narrative by Bhatia et al. [2], all 14 publications reported pain reduction following RF procedures, with pain scores decreasing by 30% to over 90% from baseline. In the narrative review by Cheney et al. [5], the nine studies demonstrated a 30%-80% reduction in pain scores over follow-up durations ranging from three months to three years. In the case reports by Kawaguchi et al. [7], 12 out of 14 patients experienced at least 50% pain relief for 1-11 months. However, few studies used validated measures of hip function (e.g., WOMAC, Harris Hip Score, and Oxford Hip Score) to assess functional improvement [2]. Two main causes of failure can be identified: axonal regeneration (as long as the nerve ganglion remains intact, regeneration occurs) and a lack of consensus on the arrangement and anatomical location of sensory articular branches around the hip joint [8]. In our case, the advanced stage of the disease also contributed to the distortion of anatomical landmarks. Paresthesias, which were transient, are the most commonly reported complications [5]. Quadriceps paralysis due to femoral neuropathy, the most severe complication, was observed in only one study [5]. Motor tests must be negative to prevent this complication [6]. Despite these risks, RF appears to be a safe technique, which is a significant advantage.

## Conclusions

RFA of the articular branches for managing hip joint pain has shown potential, but its application is limited by the lack of extensive studies. In advanced stages of conditions such as osteonecrosis, osteoarthritis, or tumors, where anatomical landmarks may be distorted or lost, the technique’s effectiveness can be compromised. Given the challenges encountered in this case, RFA should be approached with caution in advanced cases, and alternative pain management strategies should be considered. Further research is needed to develop comprehensive guidelines and refine procedural techniques for improved accuracy and outcomes.
